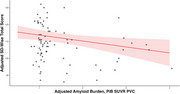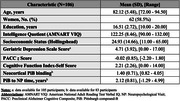# Association of wisdom and AD pathologic burden in cognitively unimpaired older adults

**DOI:** 10.1002/alz70857_099065

**Published:** 2025-12-24

**Authors:** Benjamin S Zide, Jessa Burling, David H. Adamowicz, Soyoung Lee, Keith A. Johnson, Reisa A. Sperling, Dorene M. Rentz, Nancy J Donovan

**Affiliations:** ^1^ Brigham and Women's Hospital, Boston, MA, USA; ^2^ Massachusetts General Hospital, Boston, MA, USA; ^3^ The Broad Institute of MIT and Harvard, Cambridge, MA, USA; ^4^ Harvard Medical School, Boston, MA, USA; ^5^ Center for Alzheimer Research and Treatment, Brigham and Women's Hospital, Boston, MA, USA; ^6^ Massachusetts General Hospital, Harvard Medical School, Boston, MA, USA

## Abstract

**Background:**

Wisdom is an integrated cognitive and socioemotional capacity underlying everyday decision‐making. A time‐honored construct, wisdom represents superior mental functioning that may or may not be related to age, education, intelligence quotient (IQ), or brain pathologic burden in preclinical Alzheimer's disease (AD). Leveraging an ongoing cohort study of cognition and aging, we measured wisdom and its subdomains (decisiveness, emotional regulation, self‐reflection, pro‐social behavior, social advising, tolerance for divergent values, spirituality) to determine its association with demographic and clinical variables and AD neuroimaging biomarkers in preclinical AD.

**Method:**

One‐hundred and six cognitively unimpaired (CU) older adults from the Harvard Aging Brain Study underwent assessments of self‐reported wisdom (SD‐Wise Scale), IQ (American National Adult Reading Test–Verbal), subjective and objective cognition (Cognitive Functioning Index‐Self [CFI]; Preclinical Alzheimer's Cognitive Composite [PACC]), and depression (Geriatric Depression Scale [GDS]). PiB‐PET measures of cortical Aβ were available for eighty‐three participants. Unadjusted associations between wisdom and other variables were evaluated using Pearson correlations. Separate, cross‐sectional linear models with backwards elimination estimated the associations of wisdom with each of the main predictors (PiB‐PET, CFI, or PACC). Age, sex, years of education, IQ, socioeconomic status, and GDS were included in the initial pool of predictors for each model.

**Result:**

Table 1 presents sample characteristics. Greater wisdom was correlated with female sex (t[df]=3.8[101.8], *p* <0.001) and lower GDS scores (*r* = ‐0.4, *p* <0.001), but not with age, socioeconomic status, years of education, or IQ. In the final linear model, greater total wisdom was associated with lower PiB‐SUVr (β=‐3.51, *p=* 0.017, R^2^ =  0.28; Figure 1), female sex, and lower GDS. In analogous models, neither CFI nor PACC was associated with wisdom. In secondary models, higher PiB‐PET was associated with lower decisiveness, self‐reflection, and social advising domains of wisdom, while higher GDS was associated with lower decisiveness, emotional regulation, pro‐social behavior, and spirituality.

**Conclusion:**

Wisdom, a traditional, higher‐order construct of mental functioning, was not related to education, IQ, or subjective or objective cognition in this sample of CU older adults. Wisdom encompasses qualities of decisiveness, self‐reflection and social intelligence that may be sensitive to Aβ pathologic burden and could be a measurable, unrecognized sign of preclinical AD.